# Avian Interferons and Their Antiviral Effectors

**DOI:** 10.3389/fimmu.2017.00049

**Published:** 2017-01-31

**Authors:** Diwakar Santhakumar, Dennis Rubbenstroth, Luis Martinez-Sobrido, Muhammad Munir

**Affiliations:** ^1^The Pirbright Institute, Woking, UK; ^2^Institute for Virology, Faculty of Medicine, University Medical Center, University of Freiburg, Freiburg, Germany; ^3^Department of Microbiology and Immunology, University of Rochester Medical Center, Rochester, NY, USA

**Keywords:** interferons, innate immunity, antivirals, viruses, avian, interferon-stimulated genes

## Abstract

Interferon (IFN) responses, mediated by a myriad of IFN-stimulated genes (ISGs), are the most profound innate immune responses against viruses. Cumulatively, these IFN effectors establish a multilayered antiviral state to safeguard the host against invading viral pathogens. Considerable genetic and functional characterizations of mammalian IFNs and their effectors have been made, and our understanding on the avian IFNs has started to expand. Similar to mammalian counterparts, three types of IFNs have been genetically characterized in most avian species with available annotated genomes. Intriguingly, chickens are capable of mounting potent innate immune responses upon various stimuli in the absence of essential components of IFN pathways including retinoic acid-inducible gene I, IFN regulatory factor 3 (IRF3), and possibility IRF9. Understanding these unique properties of the chicken IFN system would propose valuable targets for the development of potential therapeutics for a broader range of viruses of both veterinary and zoonotic importance. This review outlines recent developments in the roles of avian IFNs and ISGs against viruses and highlights important areas of research toward our understanding of the antiviral functions of IFN effectors against viral infections in birds.

## Introduction

For efficient replication and spread, viruses have to breach a potent and multilayered immune system in the host. Occasionally, either due to defects in host immune responses [e.g., complement system, interferons (IFNs), and adaptive immunity] or due to successful immune-antagonism, viruses overcome these antiviral mechanisms and replicate extensively in the host. This results in the engagement of diverse cascades of cellular signaling pathways (1). One of the most potent and essential events in this host–pathogen battle is the activation of the IFN pathways (1–3).

Three classes of nucleic acid receptors are associated with the activation of the IFN pathways. The first category of intracellular pattern recognition receptors (PRRs) is the family of retinoic acid-inducible gene I (RIG-I)-like helicases (RLH), which includes RIG-I, melanoma differentiation-associated gene 5 (MDA5), and laboratory of genetics and physiology 2 (LGP2) (3). A second class of PRRs is the family of toll-like receptors (TLR) including TLR3, TLR7, and TLR9, which senses extracellular, phagosomal, or endosomal pathogen-associated molecular patterns (1). The third category of PRRs is the family of DNA sensors, which include absent in melanoma 2 (AIM2) and cyclic GMP-AMP synthetase (cGAS) (4). Upon activation, these PRRs recruit downstream signaling molecules and result, directly or indirectly, in the activation of IFN regulatory factors 3 (IRF3) and 7 (IRF7), as well as activating protein 1 (AP-1) and nuclear factor kappa B (NF-κB) transcription factors (1–4). These are minimally essential events to initiate transcription of type I IFN genes and establishment of an antiviral state by expressing hundreds of IFN-stimulated genes (ISGs) (1, 5) in infected cells.

Extensive structural and functional models have been proposed on the plasticity and dynamics of nucleic acid sensing by intracellular PRRs and on the mechanisms of IFN-induced antiviral states in mammals (1–4, 6). For a detailed description of IFN induction and other innate immune responses in mammals against viruses of diverse genetic backgrounds, we refer to other in-depth reviews (1, 2, 4, 5, 7, 8).

In this article, we offer a review of the IFN pathways and transcriptional activation of ISGs in different avian species. First, we provide an overview of the chicken IFN pathways and highlight areas that differ from mammalian IFN induction and signaling. Then, we convey a comparative genetic and genomic analysis of characterized components of IFN systems among different avian species. We conclude with a description of currently studied antiviral effectors, their implications for avian diseases, and future perspectives.

## The Chicken IFN Pathways: Sensing of Viral Nucleic Acids

### RLH-Mediated IFN Induction

**Figure 1:**
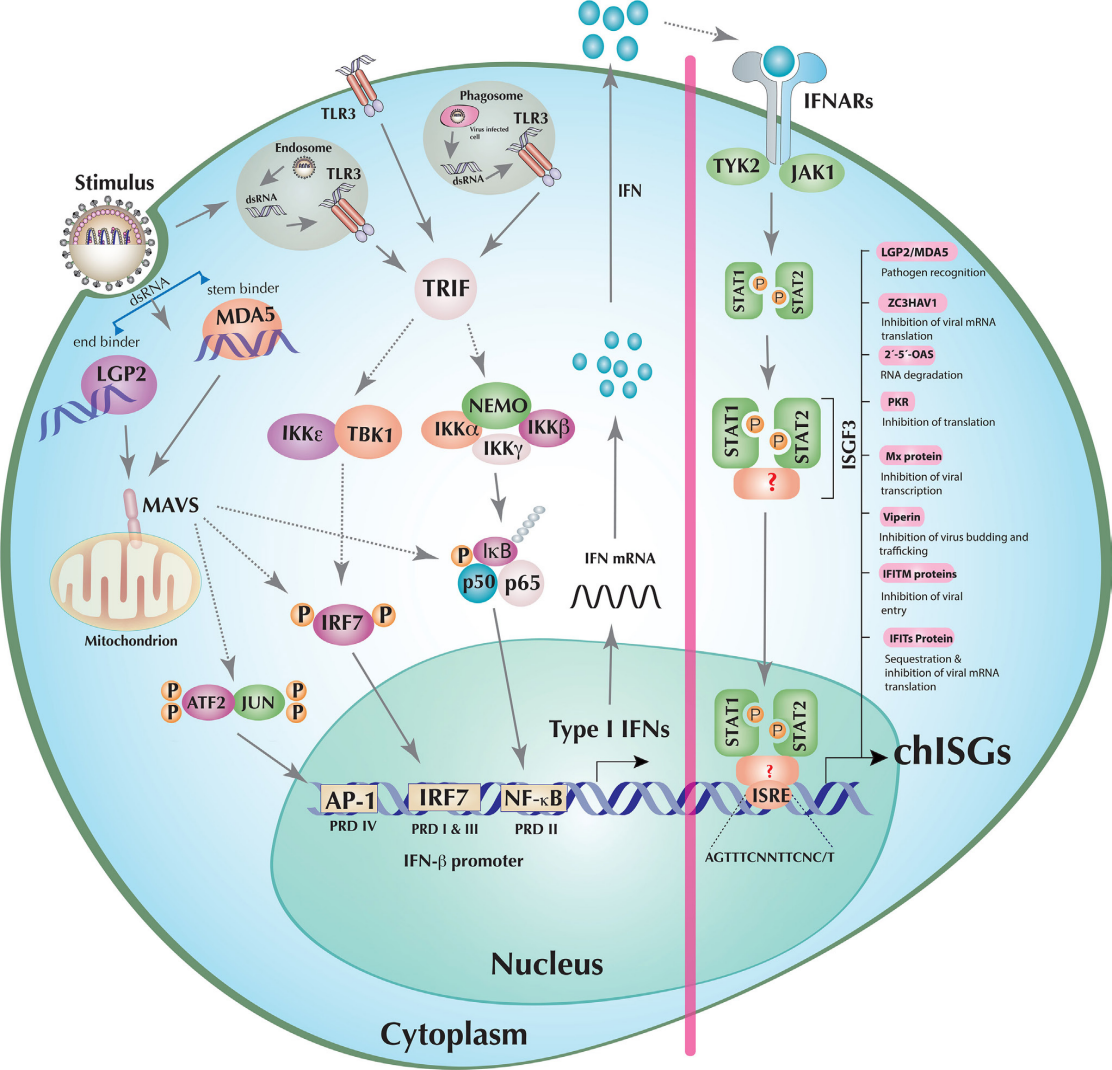
**Induction of interferons (IFNs) and establishment of an antiviral state in a model chicken cell**. The double-stranded RNA (dsRNA), detected by either chicken retinoic acid-inducible gene I (RIG-I)-like helicase (RLH) [melanoma differentiation-associated gene 5 (MDA5) or laboratory of genetics and physiology 2 (LGP2) individually or in cooperation] or toll-like receptor (TLR)3 (endosomal, phagosomal, or transmembrane) initiates downstream signaling mediated through mitochondrial antiviral-signaling protein (MAVS) or TRIF, respectively. These adaptor molecules then activate the transcription factors IFN regulatory factor (IRF)7, nuclear factor kappa B (NF-κB), and activating protein 1 (AP-1) (ATF2/JUN) by orchestrating the assembly of multi-protein complexes. Once activated, IRF7, NF-κB, and AP-1 translocate to the nucleus where they stimulate the transcription of, among others, type I IFNs (e.g., IFN-β). The transcribed, translated, and secreted type I IFNs initiate the JAK–STAT pathway by both autocrine (depicted in the figure) and paracrine signaling through cognate type I IFN receptor recognition. Activated JAK–STAT leads to the phosphorylation of STAT1 and STAT2 molecules, which (together with factors that are currently unknown in chicken) results in the formation of the IFN-stimulated gene factor 3 (ISGF3) transcription factor complex. This multifunctional transcription factor then scans and recognizes unique IFN-stimulated response element (ISRE) sequences to initiate the transcription of hundreds of chicken IFN-stimulated genes (chISGs), which subsequently establish the antiviral state against the invading viruses. Few examples of IFN-stimulated genes (ISGs) along with a summarized description of their functions are enlisted in the right panel of the figure. Abbreviations used in the figure and are not described in the main text are as follows: IκB kinase (IKK) epsilon (IKKε), alpha (IKKα), beta (IKKβ), and gamma (IKKγ); NF-κB essential modulator (NEMO); TANK-binding kinase 1 (TBK1); inhibitors of NF-κB (IκB), NF-κB subunits p50 and p65; activating transcription factor 2 (ATF2); tyrosine kinase 2 (TYK2); Janus kinase 1 (JAK1); signal transducer and activator of transcription 1 (STAT1), and STAT2. “P” represents the phosphorylation state of the protein, and dotted lines indicate the involvement of multiple intermediary steps.

The principles of mammalian IFN pathways (exemplified by humans) are in general transferable to chickens. However, there are considerable evolutionary divergences in some of the key elements of the chicken IFN responses to avian viruses if compared to their mammalian counterparts. In mammals, RIG-I primarily senses 5′-triphosphorylated blunt-ended or double-stranded RNA (dsRNA) produced during RNA virus infections. On the other hand, MDA5 can be activated by long dsRNA, whereas LGP2, which differs from RIG-I and MDA5 in lacking caspase activation and recruitment domain domain, can positively regulate MDA5 and negatively regulate RIG-I signaling (9, 10). One of the most striking features of chickens and other members of the order Galliformes (e.g., turkeys) is the absence of RIG-I (11). Despite the absence of this key PRR, chickens respond to highly pathogenic avian influenza virus (HPAIVs) and mount potent type I IFN responses, probably due to cooperative actions of MDA5 and LGP2 (10, 12, 13) (Figure 1). Additionally, unlike the mammalian MDA5, which senses only long dsRNA, it appears that chicken MDA5 can also sense short dsRNA implying that chicken MDA5 may compensate, to some extent, the function of RIG-I in chickens (13). Recently, Uchikawa et al. have resolved the structures of dsRNA-bound chicken LGP2 and MDA5 and revealed the plasticity of nucleic acid sensing by these RLH (10). It was shown that chicken LGP2 carries two properties of RLH: an MDA5-like helicase domain and a RIG-I-like C-terminal domain. Chicken LGP2, similar to human RIG-I, is an “end binder,” whereas chicken MDA5 is a “stem binder” of dsRNA (Figure 1). Based on structural (10) and functional studies (12, 13), it is likely that chicken LGP2-mediated enhancement of MDA5 sensing of dsRNA is dependent on RNA binding. However, it remains to be demonstrated if the mechanisms of LGP2-mediated enhancement of MDA5 signaling are similar to its mammalian counterparts or if the absence of RIG-I in chickens can contribute in the dynamics of cooperative nucleic acid sensing in chickens.

It has been hypothesized that the lack of RIG-I makes chickens highly susceptible to RNA viruses, and therefore chickens continue to play a central role in the emergence of zoonotic influenza viruses (14). However, more research is still required to support this generally accepted concept. Although MDA5 and LGP2 seem to be sufficient to induce a potent activation of the type I IFN pathway, ectopic expression of duck RIG-I in chicken cells potentiated the downstream signaling pathway, including increased induction of several ISGs such as myxovirus-resistance protein (Mx), protein kinase R (PKR), IFN-induced protein with tetratricopeptide repeats 5 (IFIT5), or 2′-5′-oligoadenylate synthetase (2′-5′-OAS) (14, 15). These studies indicate that chickens have acquired mechanisms to compensate the deficiency of the RIG-I signaling molecule; however, it is not possible to assess the outcome of nucleic acid sensing in chickens as it would have been in the presence of endogenous RIG-I. Nevertheless, chickens are one of the most successfully domesticated animal species and are immunologically competent in mounting an effective antiviral type I IFN state against diverse stimuli.

### TLR-Mediated IFN Induction

Toll-like receptors are type I transmembrane proteins and have a highly conserved architecture in a variety of species, including insects, fish, amphibians, birds, and mammals (16). Comparative biological approaches revealed that chicken TLRs carry unique properties regarding ligand specificity, formation of TLR receptor complexes, and activation of signaling pathways (17). At least 10 different TLR members (TLR1–10) have been identified in humans (16). Chickens have been shown to have two TLR2 isoforms (chTLR2 types 1 and 2), two TLR1/6/10 orthologs, and single genes for TLR3, TLR4, TLR5, and TLR7. Interestingly, chickens do not possess the viral DNA sensor TLR9. However, TLR-mediated DNA sensing is mediated by a functional ortholog TLR21, which is absent in humans (16). Additionally, it has been proposed that chicken TLR8 is non-functional and that the chicken genome encodes for an additional TLR gene, TLR15, which requires protease-cleavage for activation (18). Beside genomic variations, functional differences exist in the mechanism of TLR-mediated signal induction in chickens. In contrast to humans, lipopolysaccharides failed to stimulate the TLR4–TRAM–TRIF pathway in chicken cells (19). Among all mammalian TLRs, TLR3, TLR7/8, and TLR9 are known to sense viral dsRNA, ssRNA, and DNA molecules, respectively. Since in chicken TLR8 is non-functional and TLR9 is absent, only TLR3 and TLR7 are involved in the recognition of RNA viruses. All TLR family members, with the notable exception of TLR3, signal *via* myeloid differentiation primary response 88 (Myd88). TLR3 recruits TIR-domain-containing adapter-inducing IFN-β (TRIF) through transmembrane, phagosomal, or endosomal compartments (Figure 1). Both modes of TLR-dependent signal induction culminate in the activation of the transcription factors required for the transcription of type I IFNs.

### DNA Sensors-Mediated IFN Induction

In addition to TLR9-mediated DNA sensing in mammals, cytosolic DNA, which can be either non-self DNA or results from gross nuclear/mitochondrial damage, can elicit type I IFN responses in mammals (9). Currently two major cytosolic sensors of DNA have been characterized: the PYHIN family member AIM2 and cGAS. Additionally, several proteins have been recognized as DNA receptors, including Z DNA binding protein 1 (ZBP1/DAI), the helicase DDX41, and IFI16, another member of the PYHIN/HIN-200 family (20, 21). Downstream of these DNA sensors, the stimulator of IFN genes (STING) acts as an adapter and stimulates type I IFN production through the activation of IRF3 and NF-κB transcription factors (9). Although DNA sensing in chickens has not yet been explored in greater detail, genetic analysis indicate that the AIM2 gene has been lost independently in several animals, including bats and chickens (22). Even in the latest Ensembl release of the chicken genome, ZBP1 and IFI16 were not identified, suggesting fundamental differences in DNA sensing mechanisms in chickens. However, it has been shown recently that chicken STING can actively sense DNA and in cooperation with the mitochondrial antiviral-signaling protein induces type I IFN responses independent of RIG-I, interfering with the replication of RNA viruses (23). Interestingly, STING-mediated type I IFN induction was synergistically supported by RLHs in chickens (23). This warrants future investigations to understand the molecular mechanisms underlining DNA sensing in chickens.

## Transcriptional Activation of IFNs

Signals initiated by the sensing of viral nucleic acids by RLHs, TLRs, or DNA sensors lead to the activation of at least three transcription factors (AP-1, IRF3, and NF-κB) in the mammalian type I IFN enhanceosome (1). There is scarcity in our current understanding of the mechanism and structure of the chicken IFN enhanceosome. Comparative genomics analysis indicates that chickens are IRF3 deficient (detailed below). Currently, it is not known if the presence of functional IRF7 in chickens compensates for the IRF3 deficiency. Components of AP-I and NF-κB transcription factors are encoded in the chicken genome, and it is likely that these signaling cascades are functionally similar to mammals. Thus, a direct functional comparison may be plausible. While inactive, NF-κB, IRF3/IRF7 (in mammals and IRF7 in chicken), and AP-1 remain in the cytoplasm; however, upon stimulation (e.g., nucleic acids) these transcription factors get activated and subsequently translocated to the nucleus of viral-infected cells by unique mechanisms (1). The activation signals result in phosphorylation of IRF7. Conformational changes caused by this post-translational modification result in IRF7 dimerization and exposure of the nuclear localization signal (NLS) (1). This NLS mediates the nuclear translocation of IRF7 (1, 24). The inhibitor of NF-κB (IκB) retains NF-κB molecules in the cytoplasm. However, upon activation by phosphorylation, IκB undergoes ubiquitination and proteasomal degradation. Degradation of IκB exposes the NLS of NF-κB, which leads to its nuclear translocation (7). Phosphorylation of c-jun and activating transcription factor 2, two heterodimeric components of AP-1, also causes nuclear translocation (1). In the nucleus, these three transcription factors assemble in a cooperative manner to build a type I IFN enhanceosome, which binds to its respective positive regulatory domains (PRDs). IRF7, NF-κB, and AP-1 bind to PRD I/III, PRD II, and PRD IV, respectively, where they induce the transcription of type I IFNs and pro-inflammatory cytokines (TNF, IL-6, IL-1β, etc.) (25) (Figure 1). These type I IFNs lead to transcriptional activation of several hundreds ISGs to mount an antiviral state in the host (detailed below).

## Comparative Genomics and Evolution by Gene Loss

**Figure 2:**
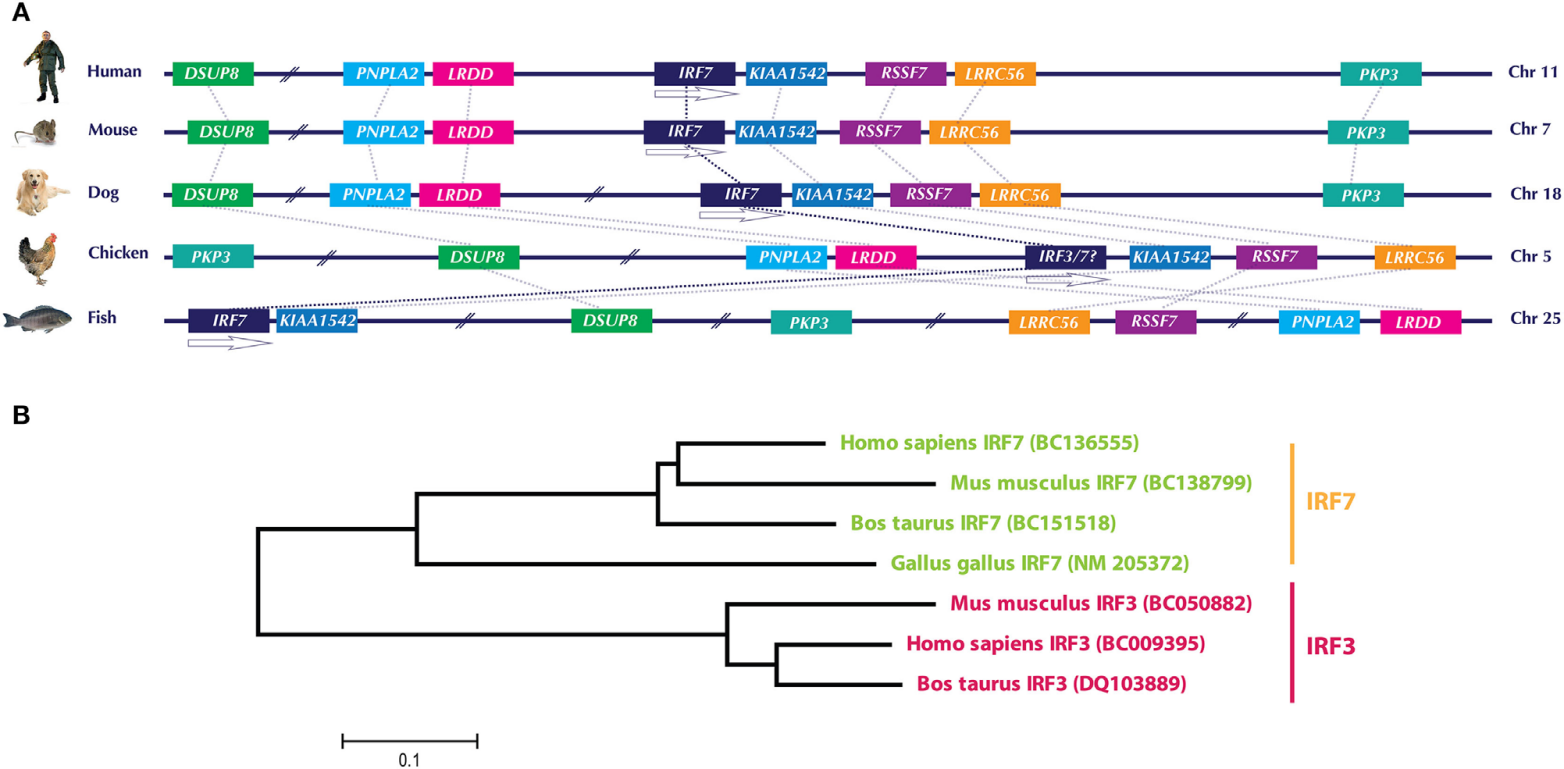
**(A)** Genomic architecture along with relative loci around the IRF7 gene in human, mouse, dog, chicken, and fish. The IRF7 genes in the compared species are flanked upstream with LRDD gene and downstream with KIAA1542 and RSSF7 genes. Direct comparison of previously identified chicken IRF3 with these species indicates that this gene is in fact IRF7. **(B)** Phylogenetic analysis of IRF3 and IRF7 genes in different species. Based on the clustering patterns and sequence homologies, the gene previously identified as “chicken IRF3” clustered closer to IRF7 of other mammals compared to mammalian IRF3. It is therefore proposed to rename “chicken IRF3” to “chicken IRF7.” Gene abbreviations used in the figure are dual specificity phosphatase 8 (DSUP8), patatin-like phospholipase domain containing 2 (PNPLA2), leucine-rich repeats and death domain containing (LRDD), interferon regulatory factor 7 (IRF7), CTD-binding SR-like protein rA9 (KIAA1542); Ras association (RalGDS/AF-6) domain family 7 (RSSF7); leucine-rich repeat containing 56 (LRRC56); plakophilin 3 (PKP3).

Even in the updated version of chicken Ensembl (Ensembl release 85—July 2016, accessed on September 11, 2016), it appears that chickens lack IRF3 and IRF9 (depicted in Figure 1), which are essential components of the type I IFN system in mammals (1). Lately, there have been substantial improvements in the genetic analysis and functional characterization of the avian type I IFN pathway, particularly in chicken. However, the annotation of the chicken genome is not yet completed, leaving open questions on the presence or absence of the mammalian homologs in avian species. Improved annotation of chicken and other avian genomes is required to unambiguously declare the presence or absence of a particular gene in the future. This fact can be exemplified by a recent analysis of IRF3/7 in the chicken genome. The first identified and characterized member in the chicken IRF family (named cIRF3) was classified as IRF3 based on its sequence and overall functional conservation with corresponding IRF3 in other species (26). Availability of updated annotation of chicken genome in the Ensembl has filled the gaps in the chicken chromosome 5, which encodes for the IRF3/7 genes and allowed to reevaluate the IRF locus in the chicken. Based on the analysis of gene loci in different species including human, mouse, dog, and fish (Figure 2A) and previous reports (25, 27), it is convincing that the formerly reported cIRF3 is actually IRF7. Furthermore, genetic clustering and sequence divergence analysis indicate that the chicken IRF7 clusters closely with IRF7 of human, mouse, and cattle compared to the IRF3 of corresponding species (Figure 2B). Therefore, it is suggested to use the term chicken IRF7 instead of cIRF3 to avoid any misunderstanding in the functional nomenclature between these two transcription factors.

**Figure 3:**
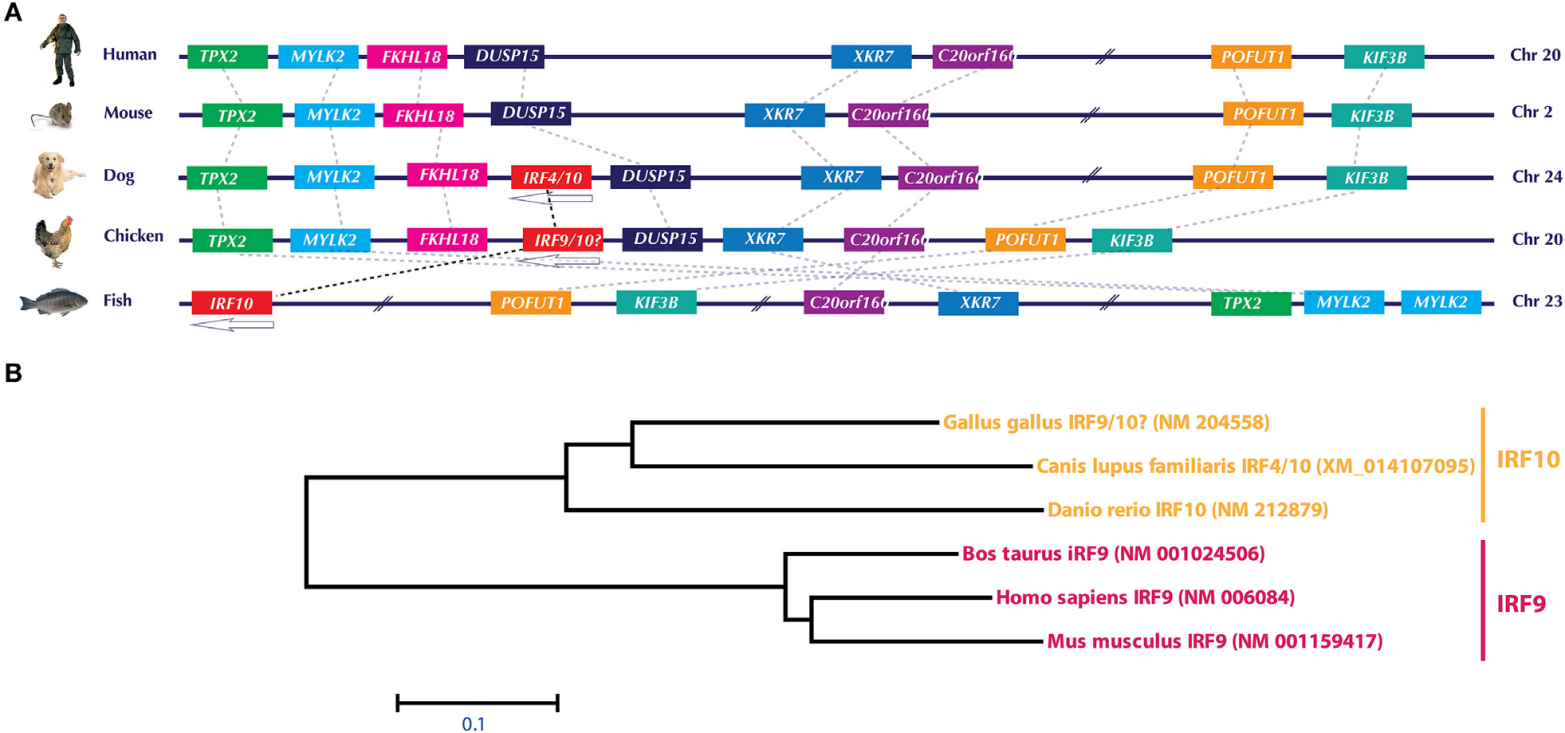
**(A)** Genomic architecture of interferon regulatory factor (IRF)10 loci and phylogenetic analysis of IRF9 and IRF10 in human, mouse, dog, chicken, and fish. Upstream and downstream genes architecture in the IRF10 of chicken, dog, and fish indicate that this locus is similar to the corresponding locus in human and mice, which lack IRF10. Based on this and phylogenetic analysis **(B)**, it is evident that the currently annotated chicken IRF9 is in fact an ortholog of IRF10. Gene abbreviations used in the figure are microtubule-associated protein homolog (*Xenopus laevis*) (TPX2); myosin, light polypeptide kinase 2, skeletal muscle (MYLK2); forkhead-like 18 (*Drosophila*) (FLKHL18); dual specificity phosphatase-like 15 (DUSP15); X Kell blood group precursor-related family member 7 homolog (XKR7); chromosome 20 open reading frame 160 (C20orf160); protein O-fucosyltransferase 1 (POFUT1); kinesin family member 3B (KIF3B).

Similar to IRF3/IRF7, the currently annotated chicken IRF9 sequence is both genetically (Figure 3A) and phylogenomically (Figure 3B) similar to IRF10 in dog and fish. Comparison of the gene orientation and architecture between species in which IRF10 is detected (dog and fish) and species in which IRF10 is lacking (human and mice) provides direct evidence that these crucial elements of the IFN pathways are currently incorrectly annotated. In addition, our analysis on global IRF family members confirms that the chicken genome lacks any significant sequence identity to the mammalian IRF9 orthologs. It remains to be explored how chickens still manage to efficiently trigger the production of ISGs without the need of IRF9 to constitute a functional type I IFN-stimulated gene factor 3 (ISGF3) complex. However, it is plausible that factor(s) other than IRF9 are involved in the formation of an active ISGF3 complex in chickens. Since type II IFNs-mediate induction of ISGs is IRF9-independent, it may be possible that under virus infection the gamma-activated sequence (GAS) promoter may overwhelm the overall induction of ISGs compared to type I and III IFN-induced expression of ISGs.

From these examples, it is clear that our understanding of the avian genome is still insufficient to accurately annotate the newly identified genes. Efforts have recently become intense through the avian consortium to not only characterize the genetics of endangered and newly sequenced bird species but also to improve the annotation of the existing genome drafts of avian species, especially chicken and duck. As a result of this, a bunch of genome sequences from more than 40 avian species was published recently (28), providing a valuable source for gene mapping. These resources would certainly advance our understanding in exploring genes, which are conserved across avian species, and to confirm existing genes. A special database (AvianBase) has been established to facilitate comparative genomics and immunogenetics in avian species (29).

Beside the fact that genes are incorrectly annotated and important genomic loci are not characterized in the avian species, it is likely that birds have evolutionary lost some genes during their domestication and subsequent division into required phenotypes (egg-laying versus meat-producing) (30). It requires extensive genetic and genomic investigations to confirm gene loss in the evolutionary process of avian species and to identify a minimum number of genes that can be readily lost from avian genomes without compromising the survivability. Although several models can be proposed, loss of genes due to “gene function bias” appears to be operative in chicken and other avian species (31). Gene function bias refers to the gene loss that is preferentially evident in a specific functional category; gene loss in gene ontology category of “immune responses” is highly probable in mammals compared to other vertebrates (32). A similar scenario can be applied to the gene loss in innate immune signaling pathways compared to other gene ontology categories in avian species mainly due to dispensable functional constraints. In this context, different type I IFN-induced proteins with tetratricopeptide repeats (IFITs), including IFIT1, IFIT2, IFIT3, and IFIT5 (33), have been described to play essential roles in nucleic acid sensing, antiviral responses, and protein translation in humans. All these functions of IFIT proteins are redundant, and thus the protein family is likely under selection constraints in chicken where only one IFIT protein (IFIT5) has been identified compared to four in human and mice (34). In summary, understanding the mechanisms and impacts of gene loss would reveal crucial evolutionary aspects of animal domestication and may highlight unexplored ways that could be exploited both for antiviral therapy and disease control.

## Evolution and Nomenclature of Avian IFNs

**Figure 4:**
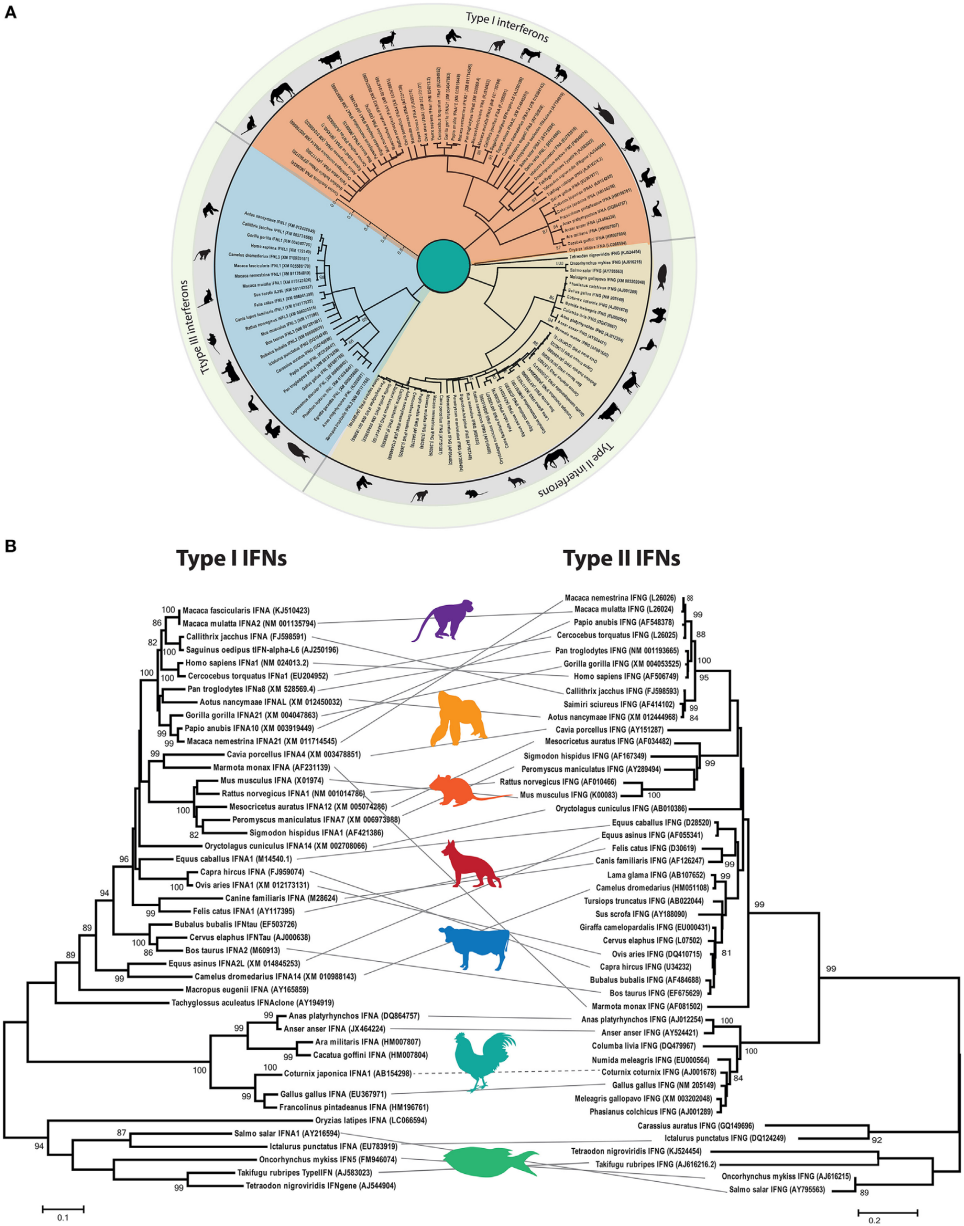
**Phylogenetic analysis of interferon (IFN) genes of mammals (including rodents, primates, and domestic animals), avian, and fish species**. **(A)** The open reading frames of type I, II, and III IFN genes were manually extracted from public databases and were aligned in BioEdit. The phylogenetic tree was constructed by MEGA6 software using the Kimura-2 model with 1,000 bootstrap replicates. The three types of IFNs clustered distantly and were labeled according to their clustering patterns. Approximate branching position was marked with a representative animal in the class. Only bootstrap values higher than 50 are shown. **(B)** Parallel comparison of type I and type II IFNs. Type III IFNs have been identified in limited numbers of species, and thus direct comparison was avoided. Clustering pattern of type I IFNs were linked to the type II IFN gene of the corresponding species for comparison purposes, and a representative animal image was shown to illustrate the clustering pattern.

Phylodynamic analysis of homology-based coding sequences of all three types of IFNs (I, II, and III) indicates that these evolutionary IFN classes are only distantly related and lack apparent sequence homology among each other (Figure 4A). However, type I and II IFNs appear to be more closely related to each other compared to type III IFN, despite the fact that type I and III share functional and signaling homologies.

Although chicken IFNs have functional homologies with their mammalian counterparts, gene duplication of each IFN subtype varies markedly among different animal species. In all birds investigated so far, type II and type III IFNs exist as a single gene each (35), whereas in mammals two to four copies of type III IFNs have been identified (35). Compared to fish, where generally only a single type I IFN homolog is detected, 3 to 10 type I IFN copies have been identified on the sex-determining Z chromosome of avian species (36–39). The maintenance of type II IFNs in avian and mammalian speciation indicates their constant function and evolutionary pressures. In both chicken and mammalian genomes, the functional transcript of the single type II IFN gene is encoded by four exons, and the gene architecture resembles that of IL-10-like cytokines.

Direct and parallel comparison of clustering patterns of type I and type II IFNs indicates the divergence of IFN-alpha (IFN-α) and IFN-gamma (IFN-γ) across mammals, rodents, primates, fish, and avian species (Figure 4B). It is evident that chicken IFNs and IFN genes of other vertebrates included in this evolutionary tree cluster distinctly from the fish IFNs. However, both fish and chicken type I and type II IFNs formed separate clades with markedly high resolution (bootstrap value of >90%). These clustering patterns may support the evolutionary and structural architecture of at least type I IFNs in different vertebrates, where fish encodes for five exons compared to single exon in birds and mammals. This is postulated to be due to the retrotransposition events in which four exons were lost between divergence of tetrapods and radiation of amniote lineages (37).

Consistent with vertebrate evolution, there are insufficient relationships between type I and type II IFNs in avian and mammalian species (Figures 4A,B). Thus, it is concluded that mammalian and avian type I IFNs evolved independently by gene duplication of a progenitor after segregation of mammals and birds (38, 39). Therefore, the avian type I IFNs are no true orthologs of their mammalian counterparts, and the nomenclature used for mammalian type I IFNs is strictly not appropriate for avian species. This is further supported by the level of genetic and functional differences between mammalian and avian type I IFNs (detailed below).

## Avian IFNs

Based on their receptor specificity, sequence homology, and nature of ISG induction, IFNs are divided into those that bind IFNαR1 and IFNαR2 (type I IFNs), those that interact with receptors complexes of IFNγR1 and IFNγR2 (type II IFNs), and those that interact with heterodimeric receptor complex of IL-28Rα and IL-10Rβ (type III IFNs or IL-28/29). Our understanding of the avian IFN pathways is gradually increasing, and recently several significant contributions have been made to characterize existing genes (40–42) and previously identified IFNs, especially in chicken. In the following sections, our current understanding on chicken IFNs and comparative genomics in other avian species will be discussed. Several known features of chicken IFNs are summarized in Table 1.

**Table 1:** **Summary of characteristics demonstrated for chicken IFNs**.

IFN type	Known variants	Chemical properties	Receptor subunits	Antiviral activities^a^	Primary expression of cytokine	Location	Promoter for ISGs	Reference
I	IFN-α, IFN-β	Acid and heat stable	IFNAR1	IFNAR2	MDV, IBDV, IBV, influenza	Fibroblasts	Z chromosome	ISRE	(43–47)
II	IFN-γ	Sensitive to low pH (2) and heat (65°C)	IFNGR1	IFNGR2	NDV, MDV, influenza	Immune cells	Chromosome 1	GAS	(48, 49)
III	IFN-λ	Heat stable	IL-28Rα	IL-10Rβ	NDV, influenza, IBV	Epithelial cells	Scaffold AADN04001262.1	ISRE	(50–52)

*^a^These are few examples of pathogens against which antiviral activities of the cytokine have been demonstrated*.

*IFN, interferon; ISGs, IFN-stimulated genes; IFN-α, IFN-alpha; IFN-γ, IFN-gamma; MDV, Marek’s disease virus; IBDV, infectious bursal disease virus; IBV, infectious bronchitis virus; NDV, Newcastle disease virus; ISRE, IFN-stimulated response element; GAS, gamma-activated sequence*.

### Avian Type I IFNs

**Figure 5:**
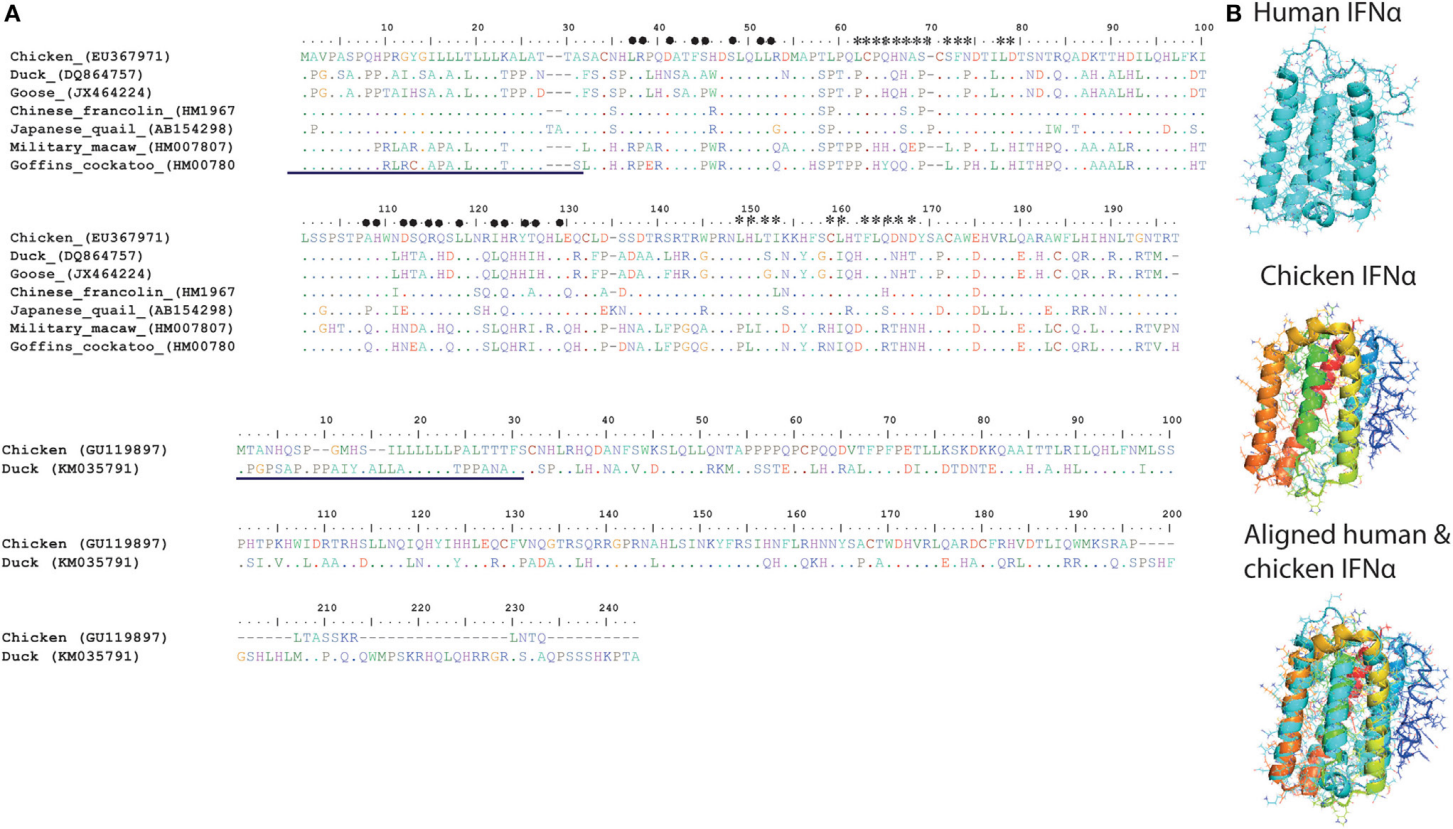
**(A)** Structural and amino acid sequence homologies between type I interferons (IFNs) in different avian species. Alignment and sequence homology of avian IFN-alpha (IFN-α) [**(A)**, top panel] and avian IFN-beta (IFN-β) [**(A)**, bottom panel] amino acid sequences. Putative sites for IFN-α binding to IFNAR1 are marked with heart symbol, whereas the sites that are important for binding to IFNAR2 are marked with star sign. In comparison to mammals, sites required for interaction of IFNs with IFNARs are more variable among avian species (54). The previously reported signal peptides are underlined in both IFN-α (top) and IFN-β (bottom) sequences (54). **(B)** A modeled cartoon structure of human and chicken IFN-α. IFN-α protein structures were predicted using I-TASSER online tool and were annotated and aligned in MacPyMOL. Similar to human IFN-α (PBD ID: 1ITF), chicken IFN-α carries five helices and is structurally similar to human IFN-α. Direct structure comparison between human (mammalian) and chicken (avian) IFN-α proteins indicate that the chicken IFN-α protein carries five alpha-helices, which are considered crucial for the functionality of type I IFNs in mammals.

In contrast to the numerous members of type I IFNs in mammals (IFN-α, IFN-β, IFN-ε, IFN-κ, IFN-ω, IFN-δ, and IFN-τ), so far only two serologically distinct, intron-less, acid and heat stable type I IFNs (IFN-α and IFN-β sharing nt homology of 57%) have been identified in avian species on the short arm of Z (sex) chromosome (53). Unlike IFN-β, which is encoded only by a single gene copy, chicken IFN-α exists as a family of several genes (Table 1). Although there is low overall amino acid identity between avian and mammalian IFN-α protein sequences (24%), a core region in the chicken IFN-α carries four of six conserved cysteine residues, an α-helix and a high sequence identity (80%) compared to mammalian IFN-α protein (Figure 5A).

Moreover, recombinant goose IFN-α has been shown to carry partial cross-species antiviral properties (55, 56). These results indicate that type I IFNs have attained certain levels of functional flexibilities (56). Nevertheless, all type I IFNs are known to be involved in inducing an antiviral state, inhibiting cell proliferation, modulating cell fate, and mediating cell differentiation and migration (57). To accomplish the primary function of IFNs, it is essential for these cytokines to bind to their respective receptors. Receptors for type I IFNs (IFNAR1 and IFNAR2) have been sequenced in chicken (58); however, little information is available about their functional domains and their crucial roles in type I IFN signaling.

Among avian type I IFNs, most of the research has been conducted on chickens, and IFN-α has been identified and more extensively characterized than IFN-β in different avian species (59) (Figures 5A,B, respectively). Chicken IFN-α and IFN-β genes were first identified from a cDNA library of aged chicken embryo cells, and subsequent analysis indicated the functionally and evolutionarily conserved properties compared to mammalian type I IFNs (60). Several recent studies have mapped the expression dynamics of chicken type I IFNs triggered by different stimuli (60–63). Collectively, type I IFNs (especially IFN-α) are potent antiviral agents and can ameliorate viral infections including Marek’s disease virus (MDV), infectious bursal disease virus (IBDV), infectious bronchitis virus (IBV), and HPAIV in different avian species (43–47). These antiviral properties of type I IFNs are identified not only *in vitro* but also *in ovo* and *in vivo* (43, 47).

Antiviral properties of type I IFNs are essentially mediated by the induction of ISGs. Both chicken IFN-α and IFN-β bind to the same IFN receptors (IFNAR1 and IFNAR2). However, it has been recently found that IFN-α and IFN-β differentially regulate ISGs in chickens (62). The antiviral state induced by chicken IFN-α was observed to be significantly more potent than that induced by chicken IFN-β, although both share genetic and structural similarities (64). These differential effects can be explained by differential binding affinity of IFN-α and IFN-β for the IFNAR1 and IFNAR2 (44). This hypothesis is further supported by a recent ontological study on the development of the chicken type I IFN system in which a markedly stronger upregulation of IFNAR1 as compared to IFNAR2 was observed during embryonic development in chicken lung and spleen cells (65). Since IFN-α and IFN-β differentially regulate the transcriptional activation of ISGs, it is imperative to consider that 5′ upstream regions of the chicken IFN-α genes lack NF-κB-binding sites and carry several binding sites for IRF members in their promoters regions (64). Moreover, observed differences in the ISGs induced by chicken IFN-α and IFN-β could be due to intrinsic functional components of the cell lines under investigation. For instance, type I IFNs induces TLR3 upregulation in the chicken fibroblasts cell line DF-1, whereas this induction was not observed in the chicken macrophages cell line HD11 (66). It cannot be excluded that constitutively primed cells may respond better to IFN-α compared to IFN-β, as has been observed in human lymphocytes that produce IFN-α, without the need to produce IFN-β, by viral infections (67). In conclusion, differential regulation of type I IFN-induced ISG signaling can be multifactorial and represents an interesting area for future investigations on the avian innate immunity.

Type I IFNs, especially IFN-α, have been characterized and assessed for their antiviral activities against IFN-sensitive viruses in various additional avian species. The duck type I IFNs were first detected in duck embryo fibroblasts (DEFs) after infection with high doses of reovirus serotype 3 (strain Dearing). Exogenous expression of this IFN blocked the release of avian RNA tumor virus particles in B77 virus-transformed DEFs (68) and showed antiviral effects for chronic hepatitis B virus infections (69). Recently, IFN-α has been identified and expressed in cells from the red-crowned crane (70), and an initial bioassay indicated its antiviral activities against vesicular stomatitis virus (VSV) in heterologous chicken fibroblasts. IFN-α has also been cloned from geese and turkeys, and initial functional insights including the antiviral actions have been determined (55, 71).

The results obtained so far on avian type I IFNs indicate that these cytokines are functionally, structurally (Figure 5B), and evolutionary related to mammalian IFNs and may have originated from common ancestor genes. However, extensive studies are required to identify other homologs of type I IFNs in all avian species, their mechanisms of action, how they exert individual and cumulative antiviral effects, and their potential for cross-species reactivity.

### Avian Type II IFNs

**Figure 6:**
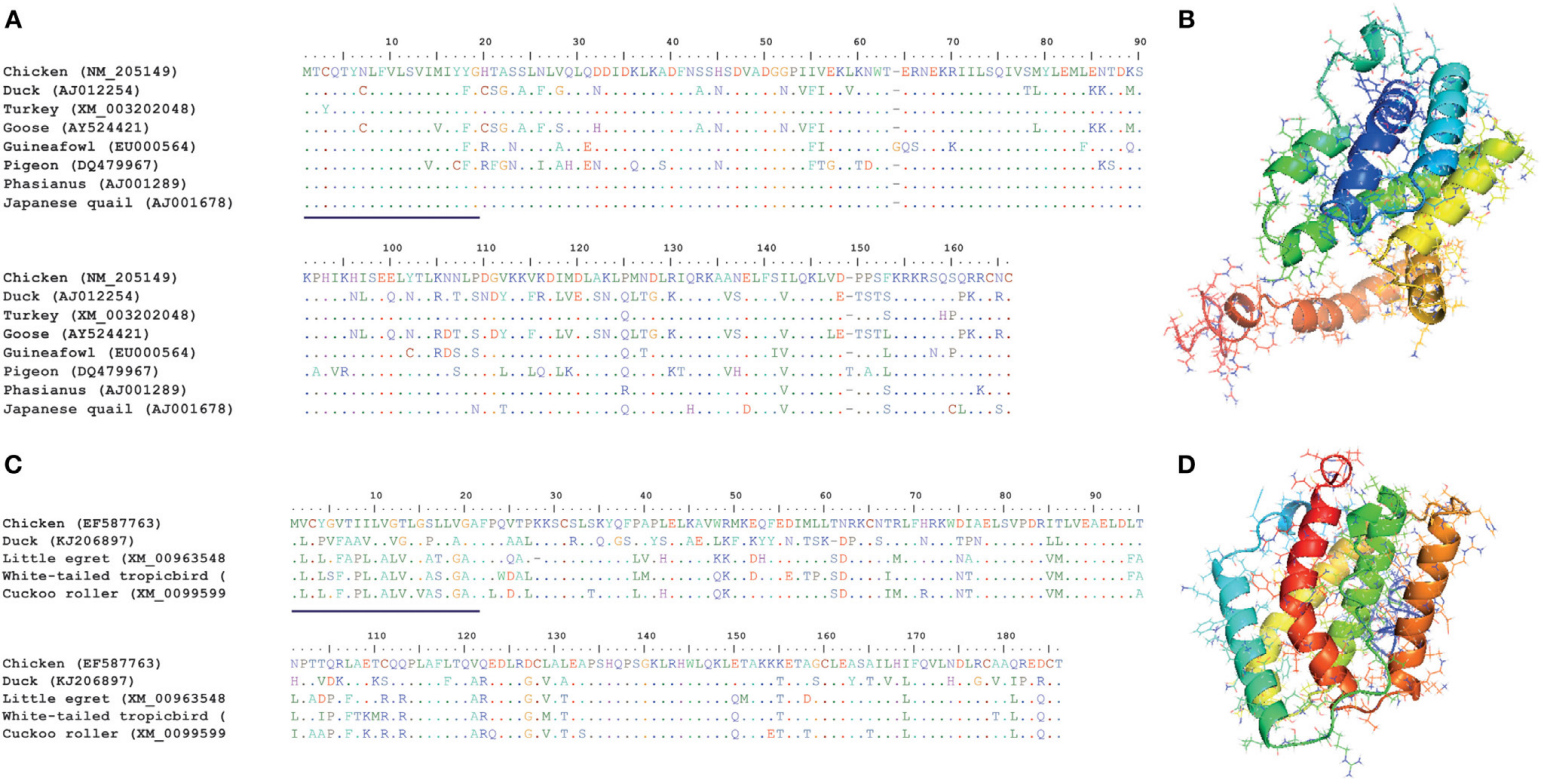
**Structural and amino acid sequence homologies between type II and type III interferons (IFNs) in different avian species**. **(A)** Protein sequence alignment of avian type II IFN (IFN-γ). **(B)** Predicted structure of chicken IFN-γ. **(C)** Protein sequence alignment of avian type III IFN (IFN-λ). **(D)** Predicted structure of chicken IFN-λ. Sequence alignments show that type II and type III IFNs are significantly conserved among avian species and may indicate interspecies cross-reactivity. Previously identified or predicted signal peptides are underlined in both IFN-γ **(A)** and IFN-λ **(C)** sequence alignments (54). These structures were predicted using I-TASSER online tool and were annotated using MacPyMOL. Structurally, these IFNs are well aligned with that of human IFNs (data not shown).

Interferon-gamma is the only member of type II IFN in birds and mammals and serves as a bridge between innate and adaptive immunity. IFN-γ plays a crucial role in regulating the maturation and differentiation process of several immune cells and activates T helper 1-type immune responses (3). Due to these unique properties, significant research has been conducted to map the antiviral potential and mechanistic effects of IFN-γ in chicken, and considerable information is also available for other avian species. Direct gene comparison and evolutionary analysis of avian IFN-γ genes clearly demonstrate the significant identity both at the genome architecture and at the core functional transmembrane domain levels (Figures 6A,B). Receptors for type II IFNs have been identified and genetically characterized in chicken (72, 73). It is interesting to observe that unlike IFN-γ receptor β-chain (IFNGR2), the IFN-γ receptor α-chain (IFNGR1) of chicken has a 110 amino acid domain of a fibronectin type III (59). The LPKS and YDKPH motifs in the intracellular domain, required for the interaction with Janus kinase 1 (JAK1) and signal transducer and activator of transcription 1 (STAT1), were found to be conserved between avian and mammalian IFNGR1 (59). From two studies conducted by the same group, it was found that chicken IFNGR1 was highly expressed in spleen, thymus, peripheral blood lymphocytes (PBLs), cecal tonsil lung, and liver, whereas chicken IFNGR2 was highly expressed in spleen, thymus, PBLs, cecal tonsil, and muscle (72, 73). Beside these fundamental investigations, our current understanding is limited to the nature and genetics of IFNGRs in avian species, which warrants extensive future research to underpin the mechanisms of the IFN-γ-induced antiviral states.

Chicken IFN-γ was first amplified from a cDNA expression library generated from a T cell line (CC8.1h) in 1995 (74). Chicken IFN-γ is encoded by a single gene located on the chromosome 1 and shares >30% amino acid homology with mammalian IFN-γ genes (74). Genetic and functional studies indicated its actions to be conserved as compared to mammalian IFN-γ-proteins (74). Unlike type I IFNs, IFN-γ is sensitive to low pH (2) and heat (65°C) (74). Several studies demonstrate that small interfering RNA mediated gene silencing of the IFN-γ to ascertain its antiviral effects (75, 76). Likewise, recent studies have clearly defined the antiviral role of IFN-γ and its adjuvant properties against viruses of diverse genetic nature including Newcastle disease virus (NDV), MDV, and influenza viruses (77–79). Similar to its mammalian counterparts, chicken IFN-γ also induces MHC class I and class II molecules and mediates the production of nitric oxide, which is an important inhibitory mechanism for viruses (80). These studies have collectively highlighted the potential and emerging roles of chicken IFN-γ in vaccine-conferred antiviral immunity.

After the initial identification of duck IFN-γ from a cDNA library generated from primary duck hepatocytes and demonstration that duck IFN-γ inhibits duck hepatitis B virus in a dose-dependent manner (81, 82), it has been found that duck IFN-γ shares both structural and functional identities with chicken IFN-γ (83). In contrast to chicken and duck IFN-γ, goose IFN-γ exerts only a weak antiviral state, which may indicate distinct biological activities between these two species (55). It is interesting to observe that the cross-species reactivity of type II IFN has been shown to be considerably higher compared to any other IFN types (48). For instance, recombinant pigeon and turkey IFN-γ was found to be functionally active in chicken cells (48, 49). In conclusion, despite of structural and functional similarities between type II IFNs in different avian species, drivers of differential antiviral activities and molecular mechanisms of diverse immunological responses induced by type II IFNs are yet to be determined in different avian species.

### Avian Type III IFN

While at least four IFN-λ genes (IFN-λ1, IFN-λ2, IFN-λ3, and IFN-λ4) were identified in humans (84), only one functionally conserved IFN-λ copy was identified in chicken (50, 51). Chicken IFN-λ shows high sequence identity with human IFN-λ3. The antiviral activities of type III IFNs are dependent on the heterodimeric IFN-λ receptor, which is composed of the IFN-λ-specific IL-28Rα (IFNLR1) chain and the IL-10Rβ (IFNLR2) chain in mammals. Expression of chicken IL-28Rα was also shown to be indispensable for the antiviral activity of chicken IFN-λ (52). Similar to mammals, expression of chicken IL-28Rα appeared to be highest on epithelial cells and in epithelium-rich organs (52). Moreover, avian type III IFN might be functionally conserved compared to those of mammalian species, likely playing a predominant role in the antiviral defense of epithelial barriers (85). This view is further supported by antiviral activity of chicken IFN-λ against several respiratory pathogens, including NDV, IBV, and influenza viruses *in vivo, in ovo*, and in epithelial cells and in tissue culture systems (52). In contrast, chicken IFN-λ showed only low to moderate antiviral effects on non-epithelial cells, such as primary chicken embryo fibroblasts (CEFs), DF-1 chicken fibroblasts, or the chicken macrophage cell line HD11 (50, 52). This is in line with the assumption that the expression of chicken IL-28Rα is low or absent in most non-epithelial cell types (52).

In contrast to chicken IFN-γ, which induces high levels of nitric oxide in immune cells, IFN-λ as well as IFN-β induces significantly lower levels of nitric oxide in different non-epithelial cell types (50). Recently, it has been shown that chicken IFN-λ inhibits influenza virus replication in CEFs; however, it requires higher doses for achieving effective antiviral activities and to induce ISGs as compared to chicken IFN-γ and IFN-β (63).

In addition to chicken IFN-λ, Yao and colleagues have recently cloned IFN-λ from Pekin ducks and have found that duck IFN-λ is genetically and structurally highly conserved to other avian and mammalian IFN-λ genes (86). Recombinant duck IFN-λ was capable of inducing ISGs (2′-5′-OAS and Mx) in primary duck hepatocytes. Only very little information is available on IFN-λ homologs in other avian species (Figures 6C,D).

## A Cross Talk Between Type I, II, and III IFNs

Following production, IFNs initiate the induction of ISGs by binding to their respective IFN receptors in autocrine and paracrine manners (1). Despite the fact that all types of IFNs play distinct and dedicated roles, a significant functional and regulatory overlap among all types of IFNs has been identified. Type I IFNs (IFN-α/β in the case of chickens) are produced from fibroblasts, whereas the antiviral actions of type III IFNs are mainly restricted to epithelial cells (52). These cell-specific roles are probably linked to the expression of cognate receptors in these organs for their importance in specific system.

It has been shown in mammals that type I and III IFNs initiate the same signaling pathway through phosphorylation of STAT1 and STAT2 heterodimers possibly by tyrosine kinase 2 (TYK2) and JAK1 kinases (1) (Figure 1). However, type II IFNs trigger ISGs’ induction *via* the activation of STAT1 homodimers by JAK1 and JAK2 kinases (1). Several protein phosphatases and the suppressors of cytokine signaling (SOCS), such as SOCS1 and SOCS3, were found to be involved in negative regulation of STATs phosphorylation (87). Although there are discrete downstream JAK–STAT signaling pathways for different type of IFNs, it has been shown that antibody-based neutralization of type I IFNs, or their receptors, attenuate the type II IFN responses. This may be linked to possible common receptor components or to the priming effect of type I IFNs on the expression of common transcription factors (e.g., STAT1) (1), which could cross-link the signaling between the three types of IFNs. Most components of JAK–STAT signaling pathway have been identified in chickens (Figure 1) and ducks, indicating possible functional homologies between mammals and avians.

To initiate the transcriptional activation of ISGs and other cytokines, type I and III IFNs mediate the recruitment and phosphorylation of IRF9 and STAT1/STAT2 heterodimer, to constitute a functional ISGF3 (1, 88). Type II IFNs initiate the formation of a STAT1–STAT1 homodimer to assemble GAF, without the need of IRF9. Upon nuclear translocation, ISGF3 and GAF bind to IFN-stimulated response elements (ISREs) (88) or GAS element, respectively (1). These events consequently lead to the transcriptional activation of hundreds of ISGs (Figure 1). In mammals, IRF9 is required for ISRE promoter activation (1). However, as indicated before, this transcription factor has not yet been identified in chickens, raising the question of alternative mechanisms of types I and III IFN-mediated ISG induction.

Regardless of the nature of their induction, ISGs play fundamental roles in a wide range of cellular activities, including transcriptional and translational regulation of immune responses (89, 90). The collective actions of these ISGs counteract viral replication and provide an antagonistic environment to limit virus propagation and spread (detailed below).

## Avian Antiviral Effectors

Binding of type I, II, and III IFNs to their respective receptors leads to the initiation of signaling cascades that culminate in the induction of distinct set of >300 ISGs (at least in human, mouse, and rats) (1, 5). These ISGs create an antiviral state and safeguard the host with multilayered, often synergistic, and cumulative actions (91). ISGs act on several stages of the viral replication cycle, ranging from virus entry to virus release (91). Some of these ISGs are PRRs that potentiate virus detection and thus modulate IFN induction through an amplification loop resulting in enhanced IFN production and hence more efficient virus inhibition (1, 91). Some ISGs have direct antiviral roles by acting at the level of host protein translation, post-transcriptional, and post-translational modifications. Significant advancements have been made in screening and mapping the antiviral roles of many ISGs against a broad range of viral pathogens (5). However, similar investigations have just been started in avian species. High throughput host gene expression profiling strategies, such as next-generation sequencing and microarray transcriptome analysis, have provided a snapshot of the ISGs that might have essential roles against avian viruses (92, 93). While the majority of these identified ISGs are still uncharacterized, a comparative knowledge of chicken/avian ISGs with their mammalian counterparts indicates that some of these ISGs are genetically and functionally conserved and are likely crucial for the control of viral infections. Of the hundreds of ISGs identified in mammals, only few have been genetically and functionally characterized in chicken. These include IFN-inducible transmembrane protein (IFITM)3 (94), which can inhibit virus entry; Mx (95), which can block early stages of virus replication; viperin (40), which can inhibit virus release; ZAP (41), which can weaken viral mRNA translation; 2′-5′-oligoadenylate synthetases (2′-5′-OAS/RNaseL) (54), which can cleave viral RNA transcripts; and PKR (62), which can sense TLR-mediated immune responses (Figure 1). To our knowledge, these are the only ISGs that have been, to date, functionally characterized in chickens. A brief description of individually known avian ISGs is provided below.

### CCCH-Type Zinc Finger Antiviral Protein (ZC3HAV1)

The antiviral action of ZC3HAV1 (ZAP) in mammals is mediated by its specific binding to the ZAP-responsive element encoded within viral mRNA (96). This binding recruits the host cellular degradation machinery to disable the viral mRNA translation specifically without any damage to host mRNA (96). Recently, chicken ZAP has been genetically characterized, and it appeared that the antiviral role of ZAP is probably evolutionarily conserved among vertebrates (41). In contrast to the presence of a long and a short ZAP isoforms in mammals, only one isoform (tentatively suggested to be the long isoform) has been found in chickens (41). The shorter isoform in mammals has recently been recognized as a positive regulator of the RIG-I pathway (97). While it remains to be finally clarified that chickens lack a shorter ZAP isoform, it may have been coevolutionary lost along with the RIG-I ortholog in chicken (41). The chicken ZAP gene can be prominently induced by polyinosinic:polycytidylic acid (poly I:C, a synthetic dsRNA analog) and type I IFN treatment in avian cells, suggesting that ZAP is an ISG (41). Moreover, the potential relevance of chicken ZAP in viral pathobiology is likely due to its upregulation in influenza H5N1 and IBDV-infected chickens (41). However, future studies are required to investigate whether all avian species have this protein and whether its functions are similar to those of its mammalian counterparts.

### IIFITM Members

Several members of the IFITM family including IFITM1, IFITM2, IFITM3, and IFITM5 have been identified in humans (34). They are differentially expressed upon stimulation by type I and type II IFNs, either in the majority of body tissues (IFITM1, IFITM2, and IFITM3) or exclusively in osteoblasts (IFITM5) (34). Recently, functions of these ISGs have been studied extensively against viruses of medical, zoonotic, and veterinary importance (34). IFITM proteins inhibit viral infection by blocking cytoplasmic entry (98). Mechanistically, IFITM proteins suppress viral membrane fusion due to reduced membrane fluidity and thus forming curvature in the outer leaflets of cell membranes (99); or by disturbing the intracellular cholesterol homeostasis by preventing association of vesicle-membrane-protein-associated protein A with oxysterol-binding protein (100). Recently, three chicken IFITM proteins (IFITM1, IFITM2, and IFITM3) have been genetically characterized, and IFITM2 and IFITM3 have been functionally characterized (94). Despite of low sequence homology, human and chicken IFITM2 and IFITM3 are functionally conversed and are potent inhibitors of influenza and lyssaviruses (94). However, it remains to be determined whether the antiviral mechanisms of chicken and mammalian IFITM members are similar. Recently, it has been demonstrated that the duck IFITM3 confers antiviral activities against influenza viruses and that this action is independent of the N-terminal region of IFITM3 (101). Interestingly, several structural divergences were observed in the duck IFITMs probably owing to host–viral coevolution. Different publically available databases clearly indicate the presence of IFITM member proteins in several other avian species with variable levels of sequence and possible functional similarities. This leaves an opportunity to identify and characterize these important effector proteins of the innate immune system and to map their functions in avians.

### Myxovirus-Resistance Proteins

Myxovirus-resistance proteins are GTPases that are key antiviral effector proteins of the type I and type III IFN pathways. In mammals, two major forms of Mx protein exist, namely MxA- and MxB-like Mx proteins (102–105). Mammalian MxA-like proteins, such as human MxA or mouse Mx1, are known to be potent inhibitors of influenza and a broad range of other viruses (102–104, 106). In contrast, the human MxB has only recently been shown to inhibit retrovirus infections (107). To date, only one lineage of Mx genes is known in birds (108, 109). Avian Mx proteins appear to be structurally similar to its mammalian counterparts, containing a GTP-binding and a leucine zipper motif, but they possess a unique N-terminal part that lacks significant homology with mammalian Mx proteins (109–111). Chicken Mx is distributed mainly in the cytoplasm (110, 112), while duck Mx has been shown to be located in cytoplasm and nucleus (111). To date, the GTPase activity for chicken Mx has not been demonstrated (113), and conflicting results have been reported on the antiviral activity of avian Mx proteins. In its first description, chicken Mx was reported to lack antiviral functions against a broad range of RNA viruses including influenza A viruses, *Thogotovirus*, VSV, and Sendai virus (110). A subsequent study identified a high degree of genetic diversity in the chicken Mx gene (114). Functional assays demonstrated that chicken Mx alleles carrying an asparagine at amino acid position 631 (Mx-Asn631) possess antiviral activity against VSV and HPAIV H5N1 in transfected mouse cells, whereas alleles carrying a serine at this position (Mx-Ser631) lacked antiviral activity (114, 115). While some studies confirmed the antiviral effects of Mx-Asn631 against VSV and NDV in cell culture (116–118), others failed to demonstrate Mx-mediated resistance of both Mx variants against influenza, NDV, and *Thogotovirus* using comparable approaches (112, 113, 119, 120). Artificial translocation of chicken Mx to the nucleus did not enhance its antiviral activity (112). *In vivo* studies either did not demonstrate an effect of the polymorphism at position 631 on the clinical course of an experimental HPAIV H7N1 infection (121) or reported an association of Mx–Asn631 with slightly reduced mortality and morbidity following HPAIV H5N2 infections of chickens (120). Overexpression of duck Mx in murine cells did not result in enhanced antiviral activity against VSV and HPAIV H7N1 (111).

In summary, the functional characteristics of avian Mx proteins, their role in innate antiviral immunity, and the effect of genetic polymorphisms are still poorly understood and require further investigations. It is possible that, similar to human MxB, avian Mx proteins possess unequivocal antiviral activities against viruses substantially differing from the few RNA virus families, which have been tested so far.

### Protein Kinase R

Protein kinase R is a serine/threonine protein kinase and consists of two domains that are functionally independent; the dsRNA-binding N-terminus and the catalytic C-terminus domains (122). PKR was first identified during investigations on the translation inhibition of viral and cellular mRNAs in vaccinia virus (VV)-infected mammalian cells (123). In an inactive form, PKR localizes in the nucleus and upon activation, mediated through viral dsRNA recognition, oxidative stress, growth factors, cytokines, and cellular proteins such as PKR-associated activator, or following the stimulation of TLRs, phosphorylates the eukaryotic initiation factor 2. This action impairs the guanine nucleotide exchange reaction and thus inhibits translation of mRNA in infected cells (124, 125). Although different viruses, including influenza virus, herpes simplex virus type I, and hepatitis C virus, encode for counteracting factors to inhibit PKR actions, this kinase can still surpass and can exert antiviral activities.

It has been demonstrated that chicken PKR carries all features characteristic for RNA-binding proteins and kinase families (126). Similar to the chicken Mx gene, chicken PKR is also polymorphic and confers antiviral effects against VSV (126). However, in an *in vivo* study, transcriptionally upregulated PKR failed to protect chickens from highly pathogenic H5N1 infection (127).

Similar to Mx and several cytokines, it is likely that specific SNPs may define the function of PKR in a specific and understudied avian population. Although PKR is one of the first identified PRRs, our understanding of its function is still incomplete even in mammals. In this regards, a novel role of PKR in specifically maintaining the integrity of newly synthesized IFN mRNAs has been recently described (128), further highlighting the need for future research (124).

### 2′-5′-Oligoadenylate Synthetase

In an attempt to understand the molecular mechanism of PKR-induced inhibition of protein synthesis during VV replication, another enzyme called 2′-5′-OAS was identified in mouse (129). Interestingly, 2′-5′-OAS mRNA has been detected in erythrocytes and immature red blood cells in several avian species (chicken, goose, and pigeon) (130, 131). The same group also identified the existence of two alleles of the 2′-5′-OAS gene in chickens (132). They found that 2′-5′-OAS A/B allele encodes for 58 and 54 kDa synthetases, whereas chickens carrying 2′-5′-OAS A/A alleles produce only a single 58 kDa protein (133). Expression of each of these two chicken 2′-5′-OAS alleles has been revealed to be age-dependent (133). The stability and persistence of 2′-5′-OAS are determined by the ubiquitin-like domain in the carboxyl-terminus of the 2′-5′-OAS (134). Interestingly, basal 2′-5′-OAS expression was systemically detected in chicken embryos independent of stimuli (130). However, a significant induction of 2′-5′-OAS was observed in IFN-treated chicken embryo cells (135).

More recently, the antiviral activity of chicken 2′-5′-OAS against West Nile virus was demonstrated in a replicon assay in mammalian cells (136). Notably, this assay provides the ability to investigate the effect of allele-specific antiviral actions of 2′-5′-OAS against avian viruses with diverse genetic backgrounds.

### Viperin

Viperin is one of the most important IFN effectors in mammals and confers antiviral activity by inhibiting the trafficking of soluble viral proteins in the cytoplasmic compartments. Limited availability of the viral components may restrict viral spread (137, 138). Moreover, several studies have also found that mammalian viperin impairs virus replication and restricted viral budding (139). The recently characterized chicken viperin exhibits mammalian-like domains, including a variable N-terminal variable region spanning 77 amino acids, a central radical SAM domain, and a C-terminal conserved region (40). While chicken viperin was significantly induced by influenza viruses and IBDV as well as by different innate immune receptor ligands both *in vitro* and *in vivo* (40), its antiviral potential requires future investigations. Since chicken viperin carries leucine zipper and radical SAM motifs, which are known to be essential for viperin-induced antiviral activities in mammals, it is conceivable that chicken viperin has functional conservation with the mammalian counterpart.

## Conclusion and Future of Avian Innate Immunity Research

The currently available information on the immunogenetics of avian IFNs is a basis for future research aimed to understand the molecular mechanisms of IFN induction, associated factors, and to identify uncharacterized IFNs in different avian species, which differ significantly in their IFNs pathways and harbor viruses of both veterinary and medical importance. Because of existing functional and genetic differences, it might be needed to revise the nomenclature of avian IFNs to truly represent their origins and actions. Although IFNs were discovered by Isaacs and Lindenmann in chicken cells (140), knowledge on the dynamics and plasticity of chicken IFNs and their antiviral activities is markedly scarce compared to their mammalian counterparts. An important and evolutionary crucial area of research is to understand the potent innate immune responses in chicken in the apparent absence of essential components of IFN pathways, such as RIG-I, especially in chicken and turkey. Recent availability of genomics data on different avian species has significantly advanced comparative immunogenetics studies. However, extensive efforts are required to improve the current genome annotation of widely used poultry species (chicken, duck, and turkey) and to effectively characterize existing gaps in functionally important genomic loci. Investigations on functional implications of avian ISGs have been started; however, next-generation strategies would be required to map the antiviral or possible proviral roles of these IFN effectors. Most actions of ISGs have been studied using single isoforms of the ISGs exploiting either ectopic expression or silencing methods. Approaches such as CRISPR/Cas9 knockout/knockin will be required for future investigations on effective mapping avian ISG and their functions. One of the aspects that might require future efforts is to identify the overlapping antiviral roles of ISGs and the molecular combinatorial networking in these antiviral, or proviral, properties. Since silencing of individual ISGs leads to observable differences in virus pathobiology, these appear to be valuable targets for the development of potential therapeutics for a broader range of viruses, and for vaccine production. In this regard, human IFNs have been successfully applied for the treatment of virus-induced human diseases; however, the clinical potential of chicken or other avian IFNs has not yet been exploited. These applications may hold options for future economical antiviral therapy not only in commercial poultry but also in companion birds.

## Author Contributions

Conception and writing of the manuscript: MM; restructuring and improvement of the contents: DR, LM-S, and DS; and designing the figures: MM and DS.

## Conflict of Interest Statement

The authors declare that the research was conducted in the absence of any commercial or financial relationships that could be construed as a potential conflict of interest.
